# Nonanatomical reduction of femoral neck fractures in young patients treated with femoral neck system: a retrospective cohort study

**DOI:** 10.1186/s12891-023-06551-2

**Published:** 2023-05-24

**Authors:** Qilong Jiang, Yang Liu, Xinwen Bai, Yu Deng, Yong Cao, Chengxiang Yu, Qizhi Song, Yan Li

**Affiliations:** 1 Department of Orthopaedic Surgery, Chongqing Orthopedic Hospital of Traditional Chinese Medicine, Chongqing, China; 2 Department of Orthopaedic Surgery, Chongqing Sanbo Changan Hospital, Chongqing, China; 3 Central Sterile Supply Department, Chonggang General Hospital, No. 1, Dayan Sancun, Dadukou District, Chongqing, 400010 China

**Keywords:** Femoral neck fractures, FNS, Femoral neck system, Nonanatomical reduction, Complications

## Abstract

**Purpose:**

Negative buttress reduction should be avoided in the treatment of femoral neck fractures (FNFs) using conventional fixation. As the femoral neck system (FNS) has been recently developed and utilized widely to treat FNFs, the association of reduction quality with postoperative complications and clinical function has not been clarified. The purpose of this study was to evaluate the clinical effect of nonanatomical reduction in young patients with FNFs treated with FNS.

**Methods:**

This multicenter, retrospective cohort study included 58 patients with FNFs treated with FNS between September 2019 and December 2021. According to the reduction quality immediately following surgery, patients were classified into positive, anatomical, and negative buttress reduction groups. Postoperative complications were assessed with 12 months of follow-up. The logistic regression model was used to identify risk factors for postoperative complications. The postoperative hip function was assessed using the Harris hip scores (HHS) system.

**Results:**

At a follow-up of 12 months, a total of eight patients (8/58, 13.8%) had postoperative complications in three groups. Compared with the anatomical reduction group, negative buttress reduction was significantly associated with a higher complication rate (OR = 2.99, 95%CI 1.10–8.10, *P* = 0.03). No significant associations were found between positive buttress reduction and the incidence of postoperative complications (OR = 1.21, 95%CI 0.35–4.14, *P* = 0.76). The difference was not statistically significant in Harris hip scores.

**Conclusion:**

Negative buttress reduction should be avoided in young patients with FNFs treated with FNS.

## Introduction

To date, femoral neck fractures (FNFs) have been ranked as the main causes of disability. Most FNFs occur in the elderly resulting from low-energy falls. Contrarily, FNFs in young patients are commonly due to high-energy trauma. Several risk factors for FNFs have been identified, including raise of age, female sex, smoking history, and osteoporosis. In the management of FNFs, closed or open reduction and internal fixation are usually performed for young patients and non-displaced FNFs, while arthroplasty is for the elderly. For displaced and unstable FNFs in patients younger than 65 years of age, anatomical reduction and rigid internal fixation are deemed to be essential [1]. In 2013, the concept of “non-anatomical reduction of displaced subcapital femoral neck fractures” was first carried out by Gotfried et al. and indicated that positive buttress and anatomical reduction position are recommended, whereas negative buttress should be avoided [2]. Several studies have evaluated the clinical efficacy of different reduction methods. Negative buttress reduction was proven to be inferior compared to positive and anatomical reduction [3, 4]. However, internal fixation modalities described in previous literature were mainly conventional implant devices, including cannulated screw, dynamic hip system, sliding hip screw, and the proximal locking plate. Due to the insufficient biomechanical characteristics of these implants, the high incidence of postoperative complications including femoral head shorting and varus, implant failure, and necrosis of the femoral head were increasingly reported [1, 5]. This may lead to an underestimation of the Gotfried nonanatomical reduction theory.

The femoral neck system (FNS), which is a minimally invasive implant, has been recently developed and utilized with superior biomechanical stability [6–8]. Thus, we conducted this study to analyze the outcomes of FNFs treated with FNS. The study aimed to investigate the different efficacy between nonanatomical reduction and anatomical reduction for FNFs in young patients treated with FNS.

## Patients and methods

This study was a multicenter, retrospective cohort study. Patients were selected from three regional hospitals. Institutional review board from all institutes participating in the study approved this research. Written informed consent was obtained from all patients included in the study.

### Patient selection

**Fig. 1:**
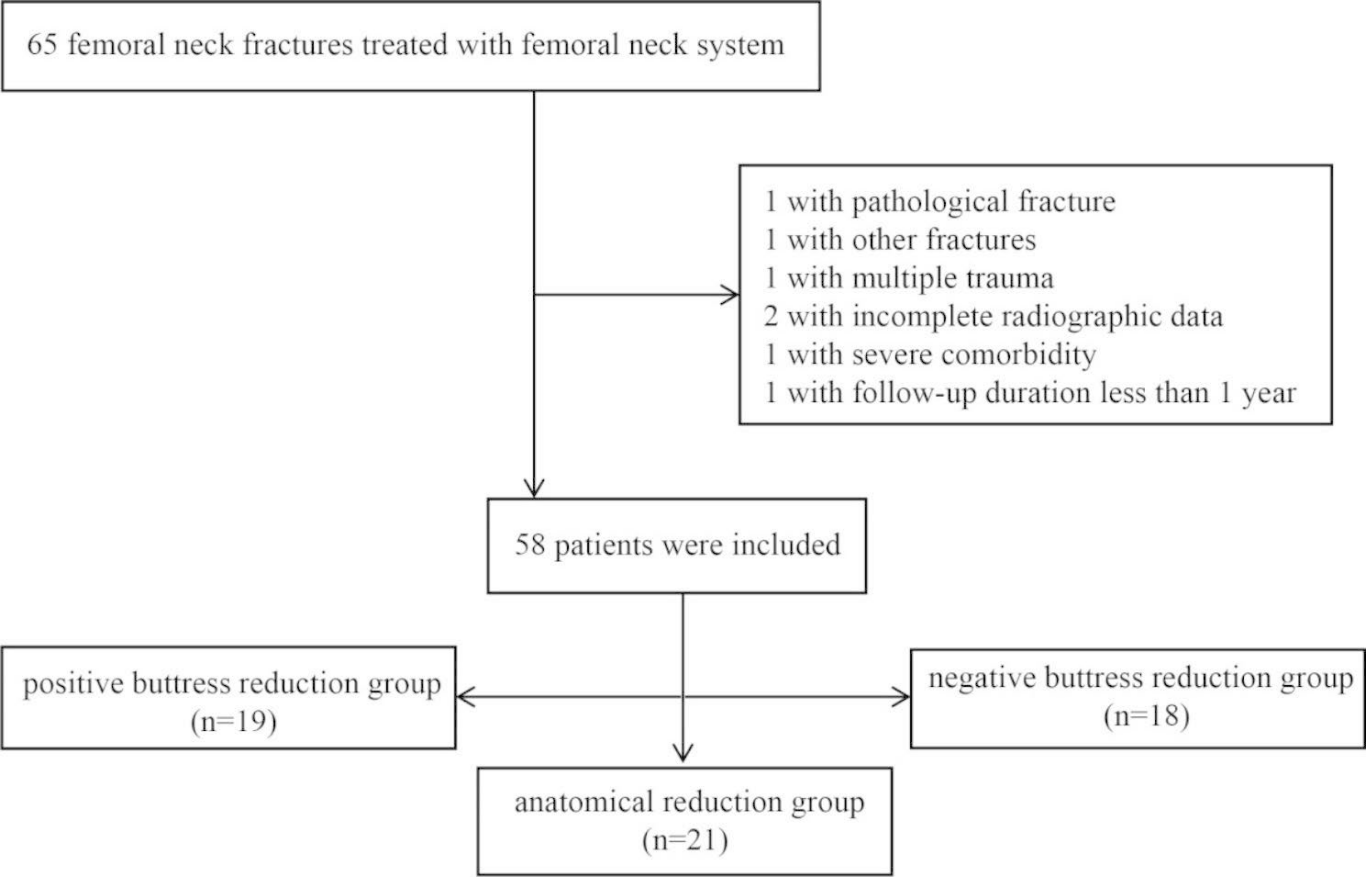
Flow diagram of included patients

Patient Selection was conducted according to the admission date between September 2019 and December 2021. Inclusion criteria were as follows: (1) The age of the young adult patients ranges from 18 to 65 years. (2) diagnosed as femoral neck fracture. (3) treated with femoral neck system. Exclusion criteria were as follows: (1) patients with pathological fractures. (2) complicating other fractures or multiple trauma. (3) incomplete radiographic data. (4) comorbidity impacting functional assessment. (5) follow-up duration less than 1 year. In total, 65 patients data were selected from the three hospitals. However, seven patients met the exclusion criteria. Finally, we included a total of 58 patients in our study (Fig. 1).

### Surgery and postoperative management

After spinal anesthesia or general anesthesia, patients were fixed in a supine position on the orthopedic traction bed. The operated limb was adjusted constantly in suitable rotation and adduction positions to obtain satisfactory reduction. A Kirschner wire was inserted across the fracture end for temporary fixation. The FNS (DePuy Synthes Products, USA) was placed following manufactory instructions. All the surgical procedures were assisted with fluoroscopic C-arm location. For FNFs of Pauwels type III or basicervical fractures, FNS with two locking holes was used. Postoperative partial weight-bearing exercises with crutches were permitted after eight weeks and usual weight-bearing activities after 12 weeks. Anterior-posterior (AP) and lateral radiographs of the affected hip were obtained immediately following surgery, one month, three months, six months, and 12 months.

### Assessment variables

**Fig. 2:**
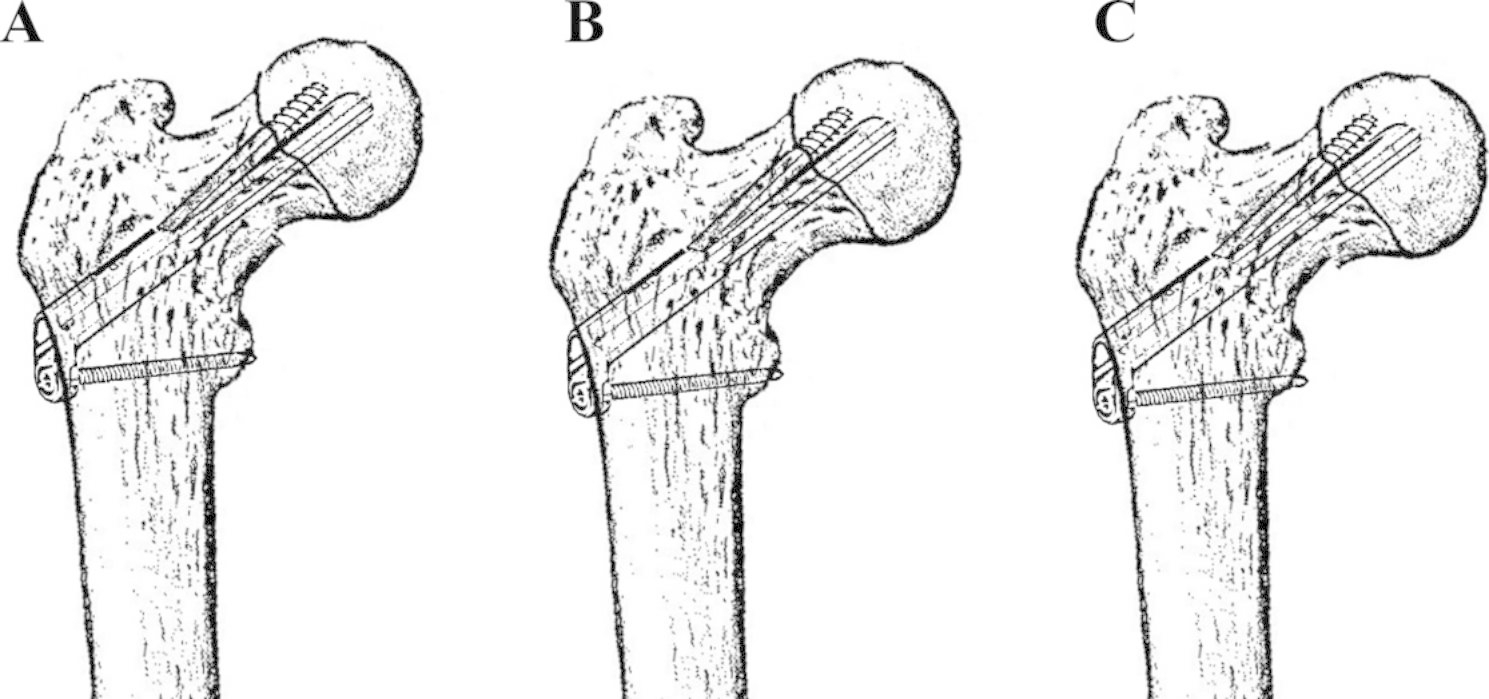
A pattern diagram showing: **A** Gotfried positive buttress reduction group. **B** anatomical reduction group. **C** Gotfried negative buttress reduction group

Based on reduction quality immediately following surgery, patients were classified into the positive buttress reduction group, anatomical reduction group, and negative buttress reduction group. The criteria of classification was based on the Gotfried concept of “nonanatomical reduction of displaced subcapital femoral neck fractures” [2]. A brief description of the nonanatomical reduction is stated in Fig. 2.

The demographics (e.g. age, gender, body mass index, smoking, and alcohol status), fracture classification, and time from injury to surgery were collected in this study. Fracture classification was performed by two blinded examiners using the Garden classification and Pauwels classification system respectively. The disagreement between the examiners regarding the type of fracture was investigated. According to previously published literature, a fracture line > 50 degrees from horizontal was regarded as a risk factor for implant failure [5]. Thus, we classified the Pauwels type into I-II and III separately.

The assessment of postoperative hip function was conducted using Harris hip scores (HHS) at 12 months of follow-up. Postoperative complications involve femoral neck shortening and varus, femoral head necrosis, nonunion, and revision surgery. The length changing of the femoral neck > 5 mm measured on AP radiographs was defined as the shortening of the femoral neck. Femoral neck-shaft angle changing more than 10 degrees postoperatively was regarded as displacement to varus. A Steinberg stage 2 or more on the postoperative radiographs was considered femoral head necrosis in this study [9]. The presence of a fracture line on radiographs one year after surgery was defined as fracture nonunion. Total hip arthroplasty was performed for Young Seniors (60–65 years of age) with implant failure, as a revision surgery. Simple implant removal surgery was excluded from the revision.

### Statistical analysis

We used SPSS software (version 25.0 SPSS Inc., Chicago, IL, USA) to take the statistical analysis. Shapiro –Wilk W test was applied to assess the normal distribution of continuous data. The means of continuous variables (e.g. Harris hip scores, follow-up time) between groups were compared using one-way ANOVA. If the difference was statistically significant, then the SNK-q test was utilized.

The Kruskal-Walllis test was used for ordinal data in groups. The chi-square test or Fisher’s exact test was used for categorical variables. The univariate and multivariate logistic regression model was used to analyze risk factors for postoperative complications. Odds ratios (OR) and a 95% confidence interval were calculated. All *P* values were two-sided and *P* < 0.05 was regarded as statistically significant.

## Results

**Fig. 3:**
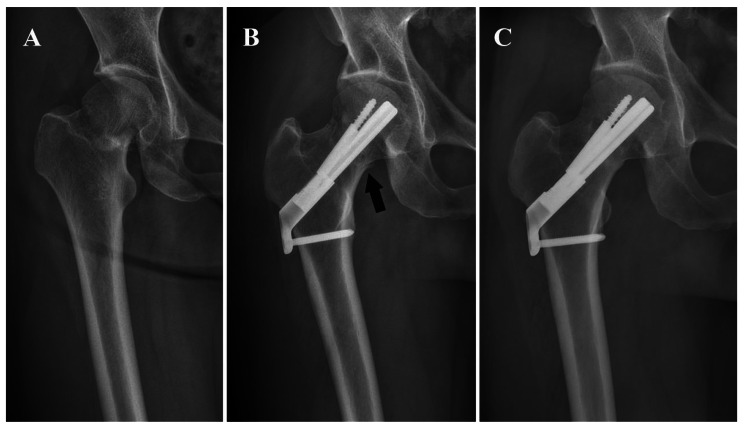
**A** Preoperative radiography. **B** Postoperative radiography and the arrow indicated positive buttress. **C** At 12 months of follow-up, the radiography showed satisfactory bone union without implant failure

**Fig. 4:**
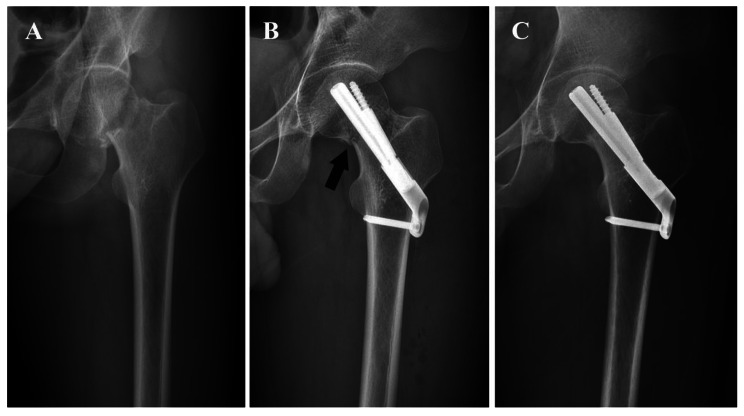
**A** Preoperative radiography. **B** Postoperative radiography and the arrow indicated negative buttress. **C** At 16 months of follow-up, the radiography showed no femoral neck shortening and varus deformity

A total of 58 patients were included, with mean age of 49.8 ± 10.7 years and mean follow-up time of 18.6 ± 9.3 months. No significant differences were noted between groups regarding baseline characteristics (Table 1). Satisfactory bone union was achieved in the majority of cases (Figs. 3 and 4). At a follow-up of 12 months, a total of eight patients (8/58, 13.8%) had postoperative complications, involving femoral neck shortening and varus, femoral head necrosis, and nonunion. Among them, four patients suffered implant failure, two of which accepted a total hip arthroplasty and achieved excellent outcomes (Table 2). Smoking status, Pauwels classification, and reduction quality were taken into multivariate logistic regression analysis to identity risk factors for postoperative complications. Smoking status (OR = 1.99, 95%CI 1.06–3.73, *P* = 0.03), Pauwels classification (OR = 2.01, 95%CI 1.22–3.33, *P*<0.01) were independent risk factors. Compared with anatomical reduction group, negative buttress reduction was significantly associated with higher complication rate (OR = 2.99, 95%CI 1.10–8.10, *P* = 0.03). No significant associations were found between positive buttress reduction and the incidence of postoperative complications (OR = 1.21, 95%CI 0.35–4.14, *P* = 0.76) (Table 3). Difference was not statistically significant in Harris hip scores (*P*>0.05).

**Table 1:** Overview of baseline characteristics

Variables	Total	Group A	Group B	Group C	*P* value
Number of patients	58	19	21	18	
Mean age ($\bar {\text{x}}$±s )	49.8 ± 10.7	50.2 ± 10.7	49.6 ± 10.9	49.9 ± 10.6	0.763
Gender					0.977
Male	31	10	11	10	
Female	27	9	10	8	
Body mass index (kg/m^2^)					
< 24	22	7	8	7	0.988
24–28	25	8	8	9	
> 28	11	4	3	4	
Smoking					0.788
Yes	11	3	4	4	
No	47	18	15	14	
Alcohol					0.881
Yes	13	4	5	4	
No	45	16	14	15	
Garden classification					0.880
I - II	35	12	13	10	
III - IV	23	7	8	8	
Pauwels classification					0.944
Pauwels I - II	47	15	17	15	
Pauwels III	11	4	4	3	
Time from injury to surgery (hours)	25.3 ± 4.4	25.6 ± 4.3	24.6 ± 5.9	25.5 ± 4.9	0.315
Follow-up time (month)	18.6 ± 9.3	18.3 ± 9.2	18.7 ± 9.1	18.5 ± 9.6	0.689

Abbreviations: Group A, Gotfried positive buttress reduction group; Group B, anatomical reduction group; Group C, Gotfried negative buttress reduction group

**Table 2:** Complications and reoperations in three groups

Variables	Total (n = 58)	Group A (n = 19)	Group B (n = 21)	Group C (n = 18)	*P* value
Postoperative complications	8	2	2	4	0.567
Shortening and varus	6	1	2	3	
Femoral head necrosis	4	1	1	2	
Nonunion	2	0	1	1	
Revision surgery	2	0	1	1	0.759
Harris hip score ($\bar {\text{x}}$ ± s )	85.9 ± 8.9	86.2 ± 8.6	85.9 ± 7.7	85.2 ± 9.1	0.836

Abbreviations: Group A, Gotfried positive buttress reduction group; Group B, anatomical reduction group; Group C, Gotfried negative buttress reduction group

**Table 3:** Multivariate logistic regression analysis of risk for complications

Variables	Adjusted OR	95%CI		*P* value
		Lower limit	Upper limit	
Smoking	1.99	1.06	3.73	0.032
Pauwels classification	2.01	1.22	3.33	0.007
Nonanatomical reduction methods				
Anatomical reduction	Reference			
Positive reduction	1.21	0.35	4.14	0.760
Negative reduction	2.99	1.10	8.10	0.032

Abbreviations: OR, odds ratios; CI, confidence interval

## Discussion

The present study demonstrated that negative buttress reduction is significantly associated with a higher rate of postoperative complications in young patients with FNFs treated with FNS, which should be avoided in the surgical procedure.

For displaced and unstable FNFs in patients younger than 65 years of age, anatomical reduction and rigid internal fixation have been regarded as the standard surgery for decades [1, 10]. Nonetheless, due to the limitation of surgical experience, anatomical reduction could not be achieved all the time [9]. Furthermore, several studies revealed that fixation with anatomical reduction may not generate impeccable outcomes. Thus, the concept of nonanatomical reduction of displaced FNFs (Gotfried Reduction) was proposed, which has been proved by several studies concerning FNFs treated with cannulated cancellous screws [2]. Specifically, according to reduction quality following surgery, nonanatomical reduction was classified into positive and negative buttress reduction. The proximal fracture fragment is located superiorly laterally to the distal fragment in the positive buttress reduction. Following gradual bone resorption and sliding between fragments, a secondary cortex support can be achieved in FNFs with positive buttress reduction. Contrarily, the proximal fragment cortex is located medially to the distal fragment in negative buttress reduction. Secondary cortex support may not occur after fragment sliding, which will lead to constant varus collapse and femoral neck shortening.

Traditional fixation alternatives for young patients with FNFs include multiple cancellous screws and sliding hip screws [1, 10]. Fixation with three parallel inverted cannulated screws shows the advantage of short operating time, minimal invasiveness, and less blood loss. Several studies have assessed clinical outcomes of nonanatomical reduction and cannulated screw fixation [2, 4, 9, 11]. Wang et al. performed a biomechanical experiment and concluded that the range of positive buttress within 3 mm could be acceptable [12]. Kai et al. surveyed 67 young patients with FNFs, which were treated with Gotfried reduction and fixation of inverted triangle cannulated screws. In this research, negative buttress reduction produced poor outcomes. Moreover, FNFs of Pauwels type III were proved to be significantly associated with a higher incidence of postoperative complications and revision surgery [11]. On the contrary, sliding hip screws have shown better biomechanical stability, albeit being more invasive [13, 14].

FNS was recently developed and applied to treat FNFs. FNS consists of three parts: the plate and locking screw, screw bolt, and anti-rotation screw. This fixed-angle system can provide compression quality, angular stability, and rotational stability [15]. The plate and screws can be placed using a minimally invasive approach. The biomechanical characteristic of FNS combines the advantages of cancellous screws and sliding hip screws, which has been corroborated by several finite element analyses. Samuel et al. performed a study including 105 FNFs treated with FNS. The rate of implant failure and the mortality rate were reported to be 13% and 21% respectively at 12 months of follow-up [8]. Amit et al. conducted a multicenter cohort study of 102 FNFs treated with FNS. Compared with traditional implants, the complication rate was significantly lower in the FNS group [16]. To our knowledge, there was no study assessing the efficacy of nonanatomical reduction and fixation with FNS. Given that the efficacy of reduction quality is still unclear in FNS-treated patients, we performed this research. In the present study, the cumulative incidence of implant failure was comparable with rates in the previous literature [16–18]. Through logistic regression analysis, smoking status, Pauwels classification, and reduction quality were defined as potential risk factors for postoperative complications. Particularly, the risk of postoperative complications in the negative buttress reduction group showed three times as high as that in the anatomical reduction buttress group. In conclusion, negative buttress reduction should be avoided in young patients with FNFs treated with FNS.

This study has certain limitations. First, the sample size was relatively small and short, which may reduce the strength of conclusions. This is mainly because the FNS has been utilized for short duration. Second, some potential risk factors were not included in regression analysis, still due to small sample size. Further research with more data is needed to validate the accuracy of relevant conclusions.

## Data Availability

The datasets used and/or analyzed during the current study are available from the corresponding author on reasonable request.

## Abbreviations

FNFs: Femoral neck fractures
FNS: Femoral neck system
AP: Anterior-posterior
HHS: Harris hip scores
